# Quantitative evaluation of a pediatric rheumatology transition program

**DOI:** 10.1186/s12969-015-0013-0

**Published:** 2015-05-24

**Authors:** Paul T. Jensen, Jill Karnes, Karla Jones, Amy Lehman, Robert Rennebohm, Gloria C. Higgins, Charles H. Spencer, Stacy P. Ardoin

**Affiliations:** Department of Internal Medicine, The Ohio State University Wexner Medical Center, 395 W. 12th Avenue Third Floor, Columbus, OH 43210 USA; Department of Pediatrics, Nationwide Children’s Hospital, 700 Children’s Drive, Columbus, OH 43205 USA; Center for Biostatistics, The Ohio State University Wexner Medical Center, 2012 Kenny Road, Columbus, OH 43221 USA; Department of Pediatrics, Cleveland Clinic Foundation, 9500 Euclid Avenue, Cleveland, OH 44195 USA

**Keywords:** (3–10) Pediatric, Rheumatology, Adolescent, Health care transition

## Abstract

**Background:**

Transition from pediatric to adult care can be a challenging process which leaves young people vulnerable to interruptions of care and worsening disease status. Efforts to improve transition processes and outcomes have included development of individualized transition plans, creation of transition clinics, and utilization of transition coordinators. Few interventions have assessed transition outcomes quantitatively.

**Methods:**

We assessed transition outcome and satisfaction of a social worker-centered transition program in a pediatric rheumatology clinic. The social worker met with patients who were 16 years or older and their families, provided transition education materials, assisted patients in developing an individualized transition plan, assisted in making appointments with an adult rheumatologist at time of transfer of care, and followed up with patients to assess transition outcomes. Patients were contacted 6–8 months after initial appointment with the adult rheumatologist to assess whether they remained in the care of the adult provider. Participants then completed a questionnaire to rate their satisfaction with the transition program.

**Results:**

210 adolescents and young adults participated in the transition program. Twenty-six similarly aged patients were eligible for transition services but did not participate in the program and were used as controls. Of the patients who participated in the program, 42 % were considered to have transitioned successfully to adult care compared to 23 % of controls (*p*-value = 0.002) of all patients. In the survey of satisfaction, 81 % of participants said that they were satisfied with the transition process.

**Conclusions:**

This study shows that a social worker transition coordinator can significantly improve the rate of pediatric rheumatology patients who successfully transition to adult care. Furthermore, patients are largely satisfied with this process.

## Background

The transition of youth from pediatric to adult care is a critical and often challenging process, particularly for those with chronic medical conditions [1–4]. Challenges which often arise during transition include: difficulty navigating new healthcare systems, gaps in insurance coverage, adolescent/young adults’ lack of health self-efficacy, and limited experience in self-management. Adolescents and young adults undergoing transition from pediatric to adult providers are often vulnerable to significant interruptions in care which can negatively impact disease status and even survival [5–8].

Recognizing the importance of improving the transition process, the American College of Physicians (ACP), the American Academy of Pediatrics (AAP), and the American Academy of Family Physicians (AAFP) developed consensus guidelines for transition which recommend development of a formal written transition process for all patients at age 14 years. These guidelines state that the transition process should be individualized and multidisciplinary in order to attend to the medical, psychosocial, and vocational needs of the emerging adult [9]. Despite such recommendations, studies report that only half of parents had ever discussed the transition process with their child’s physician and even fewer had developed a plan [10, 11]. Recent studies have aimed to measure interventions that improve the health and experience of the emerging adult as well as the costs of care [12, 13].

In rheumatologic practice the transition process is similarly difficult. A recent Childhood Arthritis and Rheumatology Research Alliance (CARRA) Survey identified that fewer than 10 % of 158 responding pediatric rheumatologists were aware of the ACP, AAP and AAFP transition guidelines and only 8 % had a formal transition processes established in their clinics [14]. A survey of parents of children with myositis identified that only 40 % of patients had help with transition related tasks [15]. A Belgian group has identified the need to strengthen communication during the transition process as well as the need for adaptation of the transition setting for the pediatric rheumatology patient [16–18]. Efforts to improve transition processes have been shown to improve the quality of life of pediatric rheumatology patients [19].

Multiple disease and specialty-specific transition models have been described in the literature. Reported models include individualized transition plans, utilization of a transition coordinator [20, 21], having adult practitioners see patients in pediatric specialty clinics [20], and establishment of a separate “transition clinic” [5, 22]. These models are often supported by descriptive data [23–29]. There are few quantitative studies regarding transition program results [2, 30]. Those that do exist often report surrogates for successful transition including patient satisfaction [20, 21, 31] though some have shown an improvement in markers of disease activity measures [32]. There is a need for additional quantitative studies to justify funding further efforts [33].

## Methods

### Patient selection

Approval was obtained from the Nationwide Children’s Hospital (NCH) Institutional Review Board for this study. For seven consecutive years, transition services were offered routinely to all rheumatology patients 16 years of age and older in the pediatric rheumatology clinic at a single, tertiary care, freestanding pediatric hospital. All patients who consented to participate in the transition program were enrolled in the study. Patients who enrolled in the study but who failed to undergo the initial assessment were used as controls.

### Transition process

At initiation of the transition process, a social worker met with each patient and family performed an assessment of transition awareness and readiness. She then provided them with a rheumatology (not disease) specific workbook that described the process of transitioning to adult care as well as lists of community, insurance, medical, and legal resources. The book also contained self-reflective questions about the patient’s views on self-care, education, occupation, hobbies, and relationships. Following completion of the workbook, the social-worker assisted the patient and family to establish written transition goals. The social worker met with patients and family at subsequent pediatric rheumatology clinic visits and by telephone to discuss progress towards transition goals and provide anticipatory guidance and interventions per the social worker’s discretion.

Transfer to adult rheumatologic care occurred when the treating pediatric rheumatologist deemed appropriate; there was no predetermined age for this to occur nor was there an upper age limit at which a patient could no longer be seen in the pediatric rheumatology clinic. The social worker facilitated the appointment with the adult rheumatologist by suggesting possible providers, providing contact information, and following up with the participants.

The social worker contacted patients by telephone or letter 6–8 months after the initial scheduled adult rheumatology appointment to ask how many times the patient had seen the adult rheumatologist. If the social worker was not able to contact the patient, the adult provider was then contacted to obtain this information. Transition success was defined as a patient having seen the adult provider twice (at least once after the initial appointment).

### Data collection

Information collected included: age, diagnosis, including age at enrollment, diagnosis, outcomes of social worker interactions, and whether the patient successfully transitioned to an adult provider. Specific information on controls was not collected other than to note whether they transitioned to seeing an adult provider.

### Satisfaction questionnaire

We created a 10 item satisfaction questionnaire for the purpose of assessing patient experience with the transition process. Responses ranged from 1 to 5 (strongly agree). The questionnaire was mailed to patients after they had first met with the adult provider and were asked to return the questionnaire by mail. Follow up phone calls were made to participants who did not return the survey.

The sum of the responses for all 10 questions was calculated to obtain a total “transition satisfaction score” ranging from 5 (most negative about transition) to 50 (most positive about the transition).

### Statistics

Baseline characteristics were summarized using descriptive statistics with categorical data presented as percentages and continuous data presented as medians and ranges. Transition status (successful, unsuccessful, and no follow-up data available) was compared between participants in the transition program and controls using a two-sided chi-square test. All analyses were performed using STAS/STAT software v9.2 (SAS Institute, Cary, NC).

## Results

**Fig. 1:**
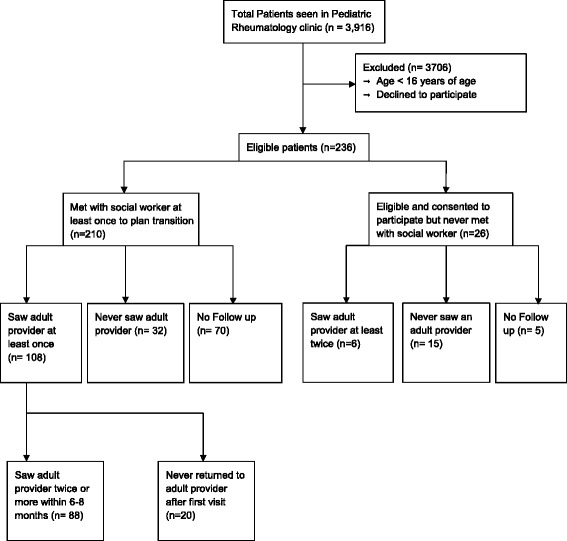
Flow sheet of participants and their involvement in the project

A total of 3916 unique patients were seen in the pediatric rheumatology clinics during this 7 year period, for a total of 18,648 visits. Out of these, 210 patients were ≥16 years of age, consented to participate in the transition program, and underwent at least the initial transition evaluation with the social worker. Twenty-six patients met the inclusion criteria but did not undergo the initial assessment and were used as controls (Fig. 1). Characteristics of the transition participants are summarized in Table 1. For both groups, the majority of patients were female with a median age of 18 years at assessment.

**Table 1:** Baseline characteristics of patients who participated in the transition program

	Participants in transition program (*n* = 210)
Female (n, %)	165 (79 %)
Age at enrollment (median, range)	18 (15–26)
Diagnoses (n, %)	
JIA – polyarticular	41 (20 %)
JIA – oligoarticular	28 (13 %)
JIA – ERA	22 (10 %)
JIA – systemic	13 (6 %)
JIA – PSA	3 (1 %)
JIA – other (JIA-NOS, not recorded)	5 (2 %)
SLE	54 (26 %)
Vasculitis	9 (4 %)
Myositis	7 (3 %)
MCTD 7 (3 %)	4 (2 %)
Other diagnoses	17 (8 %)

### Transition success

Of the 210 patients who participated in the transition program, 108 (51 %) saw an adult rheumatologist at least once. At last follow up, 88/210 (42 %) had seen an adult rheumatologist more than once and were determined to have transitioned successfully to adult services. In contrast, 20/210 (10 %) did not return after the initial visit. Additionally, 32/210 (15 %) never saw an adult rheumatologist and continued to follow with their pediatric rheumatologist; all were age 21 or younger at the time of last follow up.

Among the 26 control patients who were eligible for the transition program but did not have an initial contact and did not participate in the transition program, 6 (23 %) had a successful transition and 15 (58 %) did not. Transition information was unavailable for 5 (19 %) of these patients.

Transition outcome (successful, not successful, no follow-up available) was compared between the transition program participants and controls (Table 2). Overall there were significant differences in transition outcome between patients and controls (*p* = 0.002). A much higher proportion of control patients did not transition successfully compared to program participants (58 % vs 25 %). Conversely, 42 % of program participants transitioned successfully compared to only 23 % of controls. When those lost to follow up were combined with those who did not transition successfully, there was still a difference between the transition program group and the control group though this did not meet statistical significance (*p* = 0.06)

**Table 2:** Transition success rates

Outcome	Transition program participants (*n* = 210)	Controls (*n* = 26)	*P*-value (*n* = 26)
Transition successful	88 (42 %)	6 (23 %)	.002
Transition not successful	52 (25 %)	15 (58 %)	.002
Transition follow up data not available	70 (33 %)	5 (19 %)	.15

.

### Satisfaction survey

The response rate for the satisfaction survey (Table 3) was 57/210 (27 %). The mean satisfaction score among the 57 participants who responded to the survey was 42 (range: 16 to 50). Eighty-one percent of those who returned the satisfaction survey reported “agreed” or “strongly agreed” when asked if they were happy with the transition process.

**Table 3:** Satisfaction questionnaire

Question	Question text	Strongly disagree	Disagree	Do not agree or disagree	Agree	Strongly agree	Total
1	The transition packet I received before I transferred was a big help to me.	0	1 (2 %)	17 (34 %)	23 (46 %)	9 (18 %)	50
2	The Rheumatology Clinic staff seems to care about my future plans.	0	0	0	14 (25 %)	43 (75 %)	57
3	The biggest help I received was how to take care of myself.	0	1 (2 %)	8 (15 %)	32 (58 %)	14 (25 %)	55
4	I am happy with the transition process.	0	3 (5 %)	8 (14 %)	29 (52 %)	16 (29 %)	56
5	I feel that the Rheumatology Clinic staff just wanted to get rid of me.	42 (74 %)	13 (23 %)	1 (2 %)	1 (2 %)	0	57
6	I got the kind of help I needed to become more independent.	0	0	6 (11 %)	29 (52 %)	21 (38 %)	56
7	Rheumatology Clinic staff showed me how to get help from other places.	0	1 (2 %)	9 (16 %)	26 (46 %)	20 (36 %)	56
8	Rheumatology Clinic staff did not consider my feelings during the transition process.	39 (71 %)	12 (22 %)	3 (5 %)	1 (2 %)	0	55
9	Even if I did not want to transfer, the help I got here made me feel better about the decision.	0	1 (2 %)	8 (14 %)	24 (43 %)	23 (21 %)	56
10	I have learned a lot about how to deal with my disease on my own.	0	0	4 (7 %)	22 (39 %)	31 (54 %)	57

Satisfaction survey questions and responses. In calculations, questions 5 and 8 were reversed with more points awarded for “strongly disagree” and “disagree”

## Discussion

Transition from pediatric to adult care is recognized as critically important milestone by pediatric rheumatologists as well as the AAP, AAFP and ACP [9, 14]. Despite consensus recommendations by the AAP, implementation of formal transition processes has remained challenging [14, 15]. Our single center, social worker based transition program showed that a formal transition program is feasible and has better transition success rates compared to controls. Our measurement of young adults who follow with adult rheumatologists complements previous findings from the pediatric rheumatology literature that transition improves quality of life and ability for self-management [19]. Satisfaction with the transition process was high, though one potential, limitation is that satisfaction was measured using a tool generated for this program and not using a validated instrument.

Unfortunately, despite this formal transition program, successful transition rates remained unacceptably low at 42 % (63 % if those for whom no follow up information was available are removed.) These transition success rates are comparable to those obtained in other studies of adolescents with chronic health conditions such as diabetes and congenital heart disease [5, 6].

Despite the positive nature of this study, there are limitations. The lack of information on the controls introduces the possibility for bias. Controls were by definition patients who did not undergo the initial social-worker assessment; this leads to questions about their compliance. Additional demographic information concerning controls was not available for analysis, limiting the ability to compare to the transition group.

This study was a single center, relatively small study, and therefore results may not be generalizable to other populations. The median age at enrollment in the transition program was 18 years, older than the recommended age of 14 years [9]. Seventy-five (32 %) patients were lost to follow up. Of those not lost to follow up, only 57 (40 %) responded to the satisfaction survey which data introduces the possibility for responder bias.

## Conclusion

Despite these limitations, this study adds to the growing body of pediatric-rheumatology specific transition literature. Specifically we show that a social worker transition coordinator can improve the proportion of pediatric rheumatology patients who successfully transition to adult care.

As such, this may be considered as a transition strategy in addition to other resources [13]. Furthermore, patients are largely satisfied with the process. Our results highlight the need for improved transition processes and better transition outcomes in pediatric rheumatology, as well as the need to study quantitative outcomes of transition processes.

## Abbreviations

JIA: Juvenile idiopathic arthritis
ERA: Enthesitis related arthritis
PsA: Psoriatic arthritis
SLE: Systemic lupus erythmatosus
MCTD: Mixed connective tissue disease
